# Recombinant expression of Barnase in *Escherichia coli* and its application in plasmid purification

**DOI:** 10.1186/s12934-021-01642-y

**Published:** 2021-08-28

**Authors:** Ram Shankar, Nina Schäffer, Marco Schmeer, Joe Max Risse, Karl Friehs, Martin Schleef

**Affiliations:** 1 PlasmidFactory GmbH & Co. KG, Meisenstrasse 96, 33607 Bielefeld, Germany; 2 Fermentation Engineering, Bielefeld University, Universitätsstrasse 25, 33615 Bielefeld, Germany; 3 PlasmidFactory GmbH & Co. KG and Fermentation Engineering, Bielefeld University, Bielefeld, Germany

**Keywords:** RNase, Barnase, Plasmid, Alkaline lysis, Cation-exchange chromatography

## Abstract

**Background:**

The use of bovine-origin ribonucleases has been part of the standard protocol for plasmid DNA purification. As the field of gene therapy now enters the clinical stage, such enzymes need to be phased out or alternative purification protocols need to be developed to ensure product safety and regulatory compliance. The recombinant expression of bacterial RNase is fraught with toxicity problems making it a challenging enzyme to express. The current study describes a plasmid construct that allowed expression of barnase in *Escherichia coli* under co-expression of its native inhibitor barstar.

**Results:**

The pure enzyme without the inhibitor barstar was exported to the extracellular space through the periplasm and then purified from the cell-free supernatant. Cation exchange chromatography was employed as a primary purification step. This was followed by hydrophobic interaction chromatography which resulted in a concentrated fraction of active enzyme. Although current levels of volumetric activity achieved are quite meagre (4 Kunitz units mL^− 1^), in principle its application to plasmid DNA purification could be proved. Currently, this is capable of processing small amounts (13 g) of bacterial biomass for plasmid production.

**Conclusions:**

The current work focusses on the downstream purification strategies for a recombinant RNase and sets a framework for higher scale production if specific productivity is increased by optimal hosts and/or re-engineered plasmids. Also important is to curtail the massive enzyme loss during purification by cation exchange chromatography. Application of even a relatively small amount of recombinant RNase would contribute to greatly reducing the initial RNA levels in alkaline lysates thereby augmenting further downstream plasmid purification steps.

**Supplementary Information:**

The online version contains supplementary material available at 10.1186/s12934-021-01642-y.

## Background

Barnase (EC No. 3.1.27) produced and secreted by *Bacillus amyloliquefaciens*, is a small single-chain RNase of 110 amino acids with a molecular mass of 12.3 kDa. It contains no disulfide bonds, divalent cations as cofactors or other non-peptide moieties [1]. Barnase catalyses the cleavage of single-stranded RNA through hydrolysis of the phosphodiester bonds at GpN sites, with a higher rate for GpG and GpA sites. The activity was reported in the pH range of 7.5 to 9.3 with a maximum at 8.5 [2]. This led to the idea of expressing the protein recombinantly in its active conformation in the cytoplasm just as well as in the periplasmic space of a host cell such as *Escherichia coli* [3]. The simple structure of the enzyme also allows its active expression in a wide variety of cell types [4]. However, expression of this gene in the cytoplasm is toxic to the host cell when a corresponding inhibitor is not co-expressed. This emphasizes the significance of the corresponding intracellular inhibitor for successful recombinant expression as well [5, 6].

In *B. amyloliquefaciens*, an intracellular inhibitor of barnase called barstar is produced which tightly binds to barnase leading to inhibition and thereby protection of the host cell from the toxic effects of the RNase [4]. Barstar (89 residues), a small 10 kDa cytoplasmic protein, was the first discovered inhibitor protein specific for guanyl-specific RNases such as barnase and binase I. It binds to the active site and neutralizes any RNase that remains behind in the cytoplasm and thereby averts the danger to host cell-RNA. Due to its importance in inactivating barnase in the cell, barstar is cytoplasmic in the *Bacillus* sp. host. Barstar does not play any role in barnase expression [6]. Since the gene for barstar is not present in *E. coli*, cloning of corresponding full-length RNases is possible only when the gene for the inhibitor is cloned previously or simultaneously [5]. The inhibition mechanism has been described to be a strong non-covalent complex due to the ionic interactions between barnase and barstar. The inhibitor is described to be electrostatically optimized for tight binding [7].

Initially, the motivation for recombinant expression of barnase has been its excellent characteristics that allowed structure-function studies. In order to overcome the leaky expression from the tac promoter, the barnase gene along with a PhoA signal sequence for periplasmic targeting was cloned under the control of the λ P_R_ promoter and induced by temperature-based regulation through the c*I* repressor [8]. In later years, the motivation behind large-scale production of recombinant RNase was its application in plasmid DNA purification. For production of plasmids for gene therapy and DNA vaccines, the application of RNases is important for decrease of host cell RNA concentration in the initial purification stages. Here, the use of native bovine RNases can be avoided and the microbial recombinant alternative be used thus countering the risk of contamination with bovine spongiform encephalopathy (BSE), therefore conforming to quality standards [3].

Recombinant expression of bovine RNase A has been reported in *E. coli, Saccharomyces cerevisiae* [9, 10] and in *Pichia pastoris* [11]. Both barnase and RNase A are good candidates for recombinant expression. Barnase is coded for by a bacterial gene whereas RNase A by a eukaryotic gene. Therefore, for the latter the cDNA needs to be codon-optimized for recombinant expression in *E. coli*. Furthermore, RNase A requires the formation of four disulphide bridges. This may not be a serious limitation and therefore need not be seen as a disadvantage. Due to the reducing environment of the *E. coli* cytoplasm, the expression strategy will have to consider periplasmic expression or as inclusion bodies in the cytoplasm. Inclusion bodies necessitate refolding procedures for deriving the active enzyme but on the other hand greatly aid in initial purification of the target protein.

Both proteins have a similar length in their primary structure and similar molecular weights. Their isoelectric points are very similar (above 9) and are suitable to be purified by cation exchange chromatography. One important difference is in the mechanism of action. Barnase cleaves at GpN sites whereas RNase A cleaves at bonds 3’ from pyrimidines. Theoretically, there are more sites for action for RNase A but it has not been investigated if this leads to a higher efficiency of the enzyme.

The barnase gene along with a PelB signal sequence was cloned under the T7 promoter in a plasmid construct wherein the barstar gene had been cloned and expressed previously under its native promoter [12]. Barnase has been expressed as a model protein by fusing it to the periplasmic maltose binding protein (MBP) thus achieving secretion, and further transport into the extracellular space through co-expression of a bacteriocin release protein (BRP) [13]. Both the Sec and the Tat pathways for secretion were shown to be possible when expressed with MBP as a fusion partner with the former being more efficient. The use of MBP in this study further facilitated the application of affinity chromatography for downstream purification of excreted proteins [13].

Since the protein is toxic in its active conformation in the cytoplasm, the use of strong promoters for its expression leading to formation of inclusion bodies may actually be an advantage since inclusion bodies mostly contain large amounts of pure protein and would prevent the toxic effects of the enzyme. However, this necessitates the optimization of refolding procedures for deriving the active protein [14].

In this work the barnase gene was expressed from the P_BAD_ promoter of the arabinose operon. The PhoA leader sequence directed the enzyme (called RNase PF here) to the periplasm through the Sec pathway. A comprehensive downstream purification strategy was developed to isolate RNase activity-containing fractions by reducing the volume of cell-free supernatant several fold. Finally, experiments showing the application of the partially purified recombinant barnase in plasmid production are shown.

## Results

### Barnase expression construct

The construct PF1680 (map shown in Additional file 1) contained the barnase expression gene under the control of the P_BAD_ promoter. Based on the arabinose operon, this promoter and its regulator *araC* offered tight and efficient regulation [15]. The inhibitor barstar under the control of its native promoter was present downstream. The expression of a BRP by the *kil* gene controlled by a stationary-phase promoter increased outer membrane permeability towards the later stages of the fermentation [16]. This resulted in aided leaking of periplasmic proteins including the recombinant enzyme into the extracellular medium.

### Batch fermentation

Initial shake flask experiments and small volume batch fermentations showed the accumulation of enzyme activity in the extracellular medium. From a scaled-up batch fermentation of 20 L volume, samples of cell-free supernatants from various time points were analysed. Following induction of the barnase gene at 5 h cultivation time, the extracellular medium seems to accumulate an enzyme activity capable of degrading yeast RNA in the agarose gel assay. Some amount of DNA is seen to be released during the stationary phase (for details please refer to Additional file 2).

### Ammonium sulphate precipitation

Ammonium sulphate at 65 % saturation combined with overnight storage of supernatant at 4 °C was effective in precipitating the proteins. Following centrifugation, the proteins were resuspended in 0.01 M sodium acetate buffer (pH 5.1) to halve the volume of fermentation broth. This preparation showed a conductivity of 11.7 mS cm^− 1^. Loss of active enzyme was observed in the supernatant after centrifugation (data not shown). Also, the resulting protein fraction was high in residual salts which necessitated a desalting by ultrafiltration or by dialysis before proceeding to the chromatographic purification steps.

### Cation exchange chromatography

**Fig. 1:**
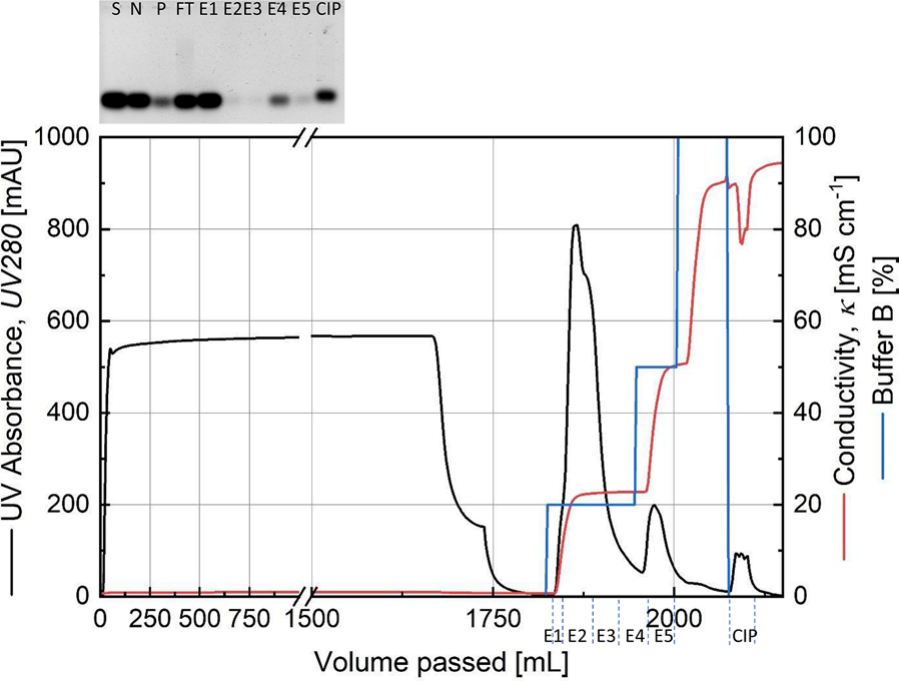
Chromatogram from a SP Sepharose FastFlow cation exchange chromatography of the retentate fraction from tangential flow filtration. Inlay: RNase activity test of various fractions. S: RNA substrate, N: Negative control, P: Permeate from TFF, FT: Flowthrough fraction, E1-E5: Elution fractions, CIP: Cleaning-In-Place fraction from cation exchange chromatography

A 20 mL bed of SP Sepharose Fast Flow packed in a XK26 column was successful in purifying the RNase activity containing fraction. In initial experiments, from among the fractions collected during a concentration gradient of buffer B, the region of 10-15 % gave a UV-peak with the best activity (data not shown). The retentate from a Tangential Flow Filtration using an Omega membrane was taken to the next downstream processing step. A fraction of the RNase enzyme activity was seen to be lost in the permeate. In the cation exchange chromatography, the purification was changed to a step-wise elution shown in Fig. 1 wherein the activity containing peak was eluted in the first step of 20 % Buffer B (fractions E2 and E3 as observed in the inlay). Other proteins were eluted in the 50 % step while a small amount of the target enzyme was also detected in E5 in this step.

**Fig. 2:**
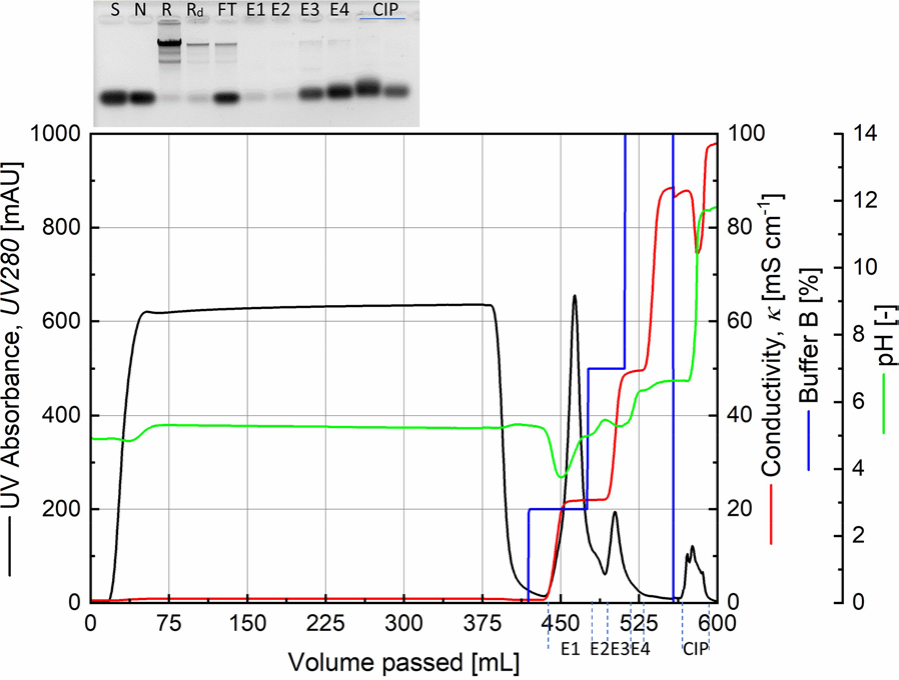
Chromatogram from a SP Sepharose FastFlow cation exchange chromatography of the retentate fraction from tangential flow filtration. The observation from Fig. 1 could be reproduced here including additional information about the buffer pH (see text for details). Inlay: RNase activity test of various fractions. S: RNA substrate, N: Negative control, R: Retentate after dialysis, R_d_: Retentate after dialysis and diluted with 10 mM sodium acetate buffer, FT: Flowthrough fraction, E1-E4: Elution fractions from cation exchange chromatography, CIP: Cleaning-In-Place fraction

The bind and elute characteristics of the target enzyme could be reproduced with the SP Sepharose FF cation exchanger medium and the 20%, 50%, 100% step-wise elution profile (Fig. 2). It was found to be beneficial to dilute the retentate from the ultrafiltration in a 1:3 ratio with the equilibration buffer to deal with problems of viscosity as well as to shift the sample pH closer to that of the equilibration buffer. The equilibration buffer was changed from 0.1 M to 0.01 M sodium acetate buffer (pH 5) in order to further reduce the conductivity during sample loading. In the agarose gel image (Fig. 2, inlay) it is seen that the retentate injected into the Äkta system contained the activity which was not lost in the flowthrough. The peak E1 and its tail E2 during the step-wise elution contained RNase activity. Relative to the retentate fed into the Äkta system, a volumetric decrease by a factor of 2.4 was achieved in the activity-containing eluted fractions.

**Fig. 3:**
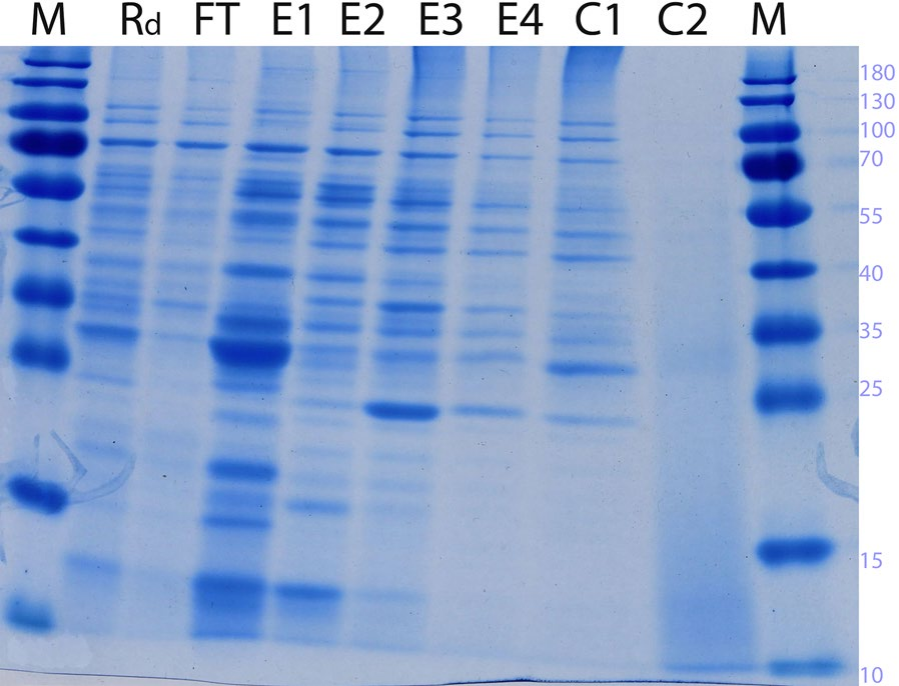
SDS-PAGE analysis of samples from the cation exchange chromatography shown in Fig. 2. C1 and C2 refer to fractions collected during Cleaning-In-Place. M: Molecular size marker with molecular weights in kDa shown

The samples were analysed by SDS-PAGE shown in Fig. 3 in which bands are observed just above the 12 kDa position for retentate, E1 and E2 which could be corresponding to the recombinant RNase PF.

An overview of the volume reduction from cell-free supernatant to the chromatography fraction is shown here (Table 1). Quantification of enzyme activity as Kunitz units in the cell-free supernatant was impeded due to high background absorbance.

**Table 1:** Accumulation of RNase activity in a reduced volume through successive steps starting from extracellular medium up to elution from a cation exchange chromatography column

Downstream stage	Cell-free supernatant	Retentate ultrafiltration	Retentate diafiltration	Activity-containing elution fraction SP Seph FF
Total protein concentration (µg mL ^− 1^)	254	1761	1332	226
Volume (mL)	7000	647	660	332
Protein amount (mg)	1778	1139	879	75

### Hydrophobic interaction chromatography (HIC)

Under the conditions tested, the chromatography medium Capto Phenyl High Sub did not result in a favourable purification of the target enzyme when fermentation broth supernatant was mixed with 3 M ammonium sulphate solution and fed into the chromatography column (data not shown). In comparison, the initial results with the medium Toyopearl 650 M Butyl showed a better purification. Elution was carried out in steps of 25%, 75% and 100% of deionized water. In the 75% step, a peak E3 was collected which showed in the agarose gel activity assay to contain RNase activity (chromatogram in Additional file 3).

### HIC as a secondary purification step

**Fig. 4:**
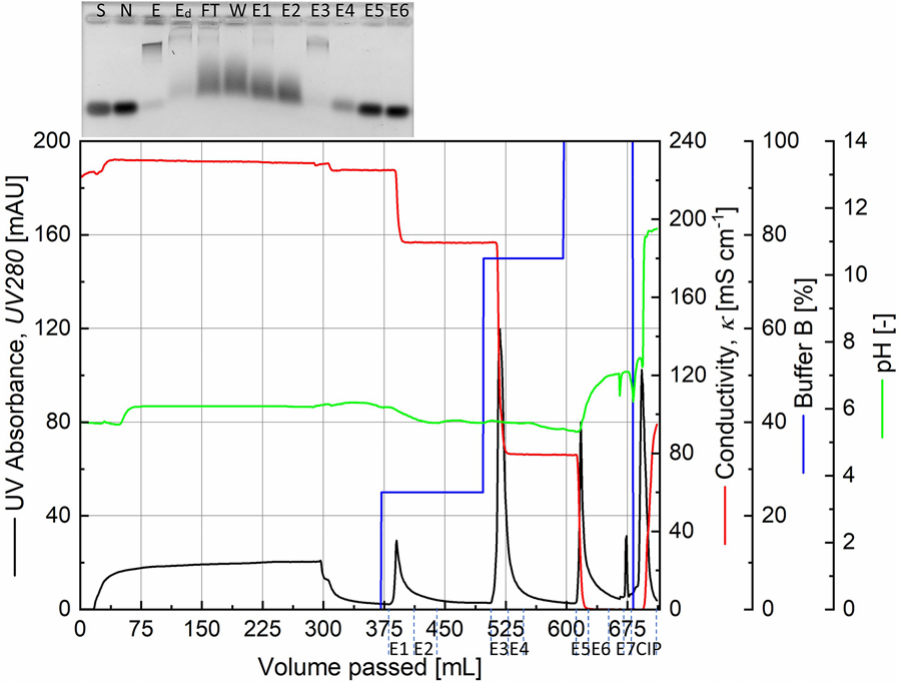
Chromatogram from a Toyopearl 650 M Butyl hydrophobic interaction chromatography of a RNase activity-containing elution fraction from a cation exchange chromatography step, prepared for HIC by mixing with 3 M ammonium sulphate. Inlay: RNase activity test of various fractions. S: RNA substrate, N: Negative control, E: Elution from cation exchange chromatography, E_d_: Elution from cation exchange chromatography diluted, FT: Flowthrough fraction, W: Wash, E1–E6: Elution fractions. The high concentration of ammonium sulphate causes smearing and RNA band displacement in the elution fractions

**Fig. 5:**
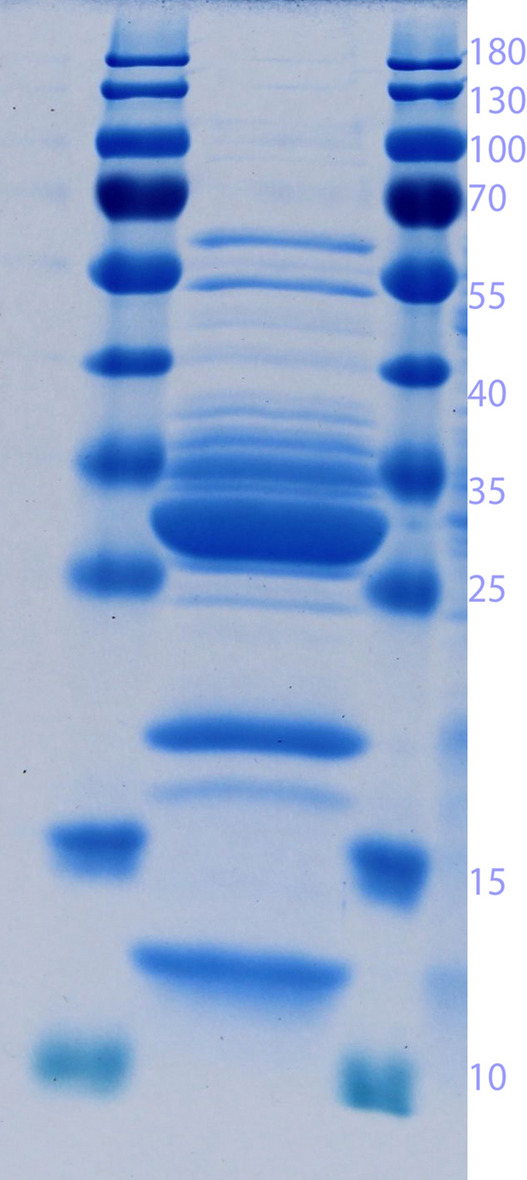
SDS-PAGE analysis of a concentrated RNase activity containing fraction (E3 of Fig. 4) from a hydrophobic interaction chromatography that followed a cation exchange chromatography purification. The lanes flanking the sample are loaded with Molecular size marker with the molecular weights in kDa shown to the right

Following the previous observation, hydrophobic interaction chromatography using the medium Toyopearl 650 M Butyl was chosen as a secondary purification step to be carried out on an RNase activity-containing eluate from the cation exchange chromatography. This result is shown in Fig. 4 where the flowthrough UV absorption is low pointing to the fact that the feed material is an eluate from a previous chromatography step with relatively few total proteins. The 75% buffer B step resulted in a highly concentrated fraction of proteins containing RNase activity as seen in Lane E3 in the agarose gel in Fig. 4. A 15-fold volumetric reduction from HIC feed to eluate was possible. The corresponding SDS-PAGE image in Fig. 5 shows the high level of concentration achieved and makes a strong case for the presence of the target enzyme at an expected 12 kDa position.

### Removal of residual plasmid DNA

A large fraction of the plasmid DNA is removed in the cation exchanger step as seen in the elution fractions in Fig. 2. In order to remove the residual plasmid from RNase PF enzyme preparations, the Sartobind range of anion exchange membrane adsorber were suitable. The activity containing fraction was loaded onto a Sartobind membrane adsorber and purified manually. The flowthrough contained the enzyme activity as well as weakly bound enzyme molecules which were recovered in a wash step. Bound DNA was eluted out separately and this fraction did not contain any RNase activity (result shown in Additional file 4).

This process was verified by qPCR using plasmid-specific primers. The enzyme samples before and after membrane adsorption were tested in different dilutions. Table 2 shows the absolute quantification of the residual DNA on the basis of a standard curve.

**Table 2:** Absolute quantification of copies of residual plasmid PF1680 in a purified RNase PF enzyme preparation. Quantification was based on a standard curve generated by amplification of standard plasmids containing the ampicillin resistance gene

Sample	Dilution	Average calculated Plasmid copy number per µL
Before Membrane adsorber	Undiluted	1.17 × 10^7^
	1:10	8.45 × 10^6^
	1:100	3.15 × 10^6^
After Membrane adsorber	Undiluted	5 × 10^2^
	1:10	5.1 × 10^2^

### Application

#### RNA removal in cleared alkaline lysate

**Fig. 6:**
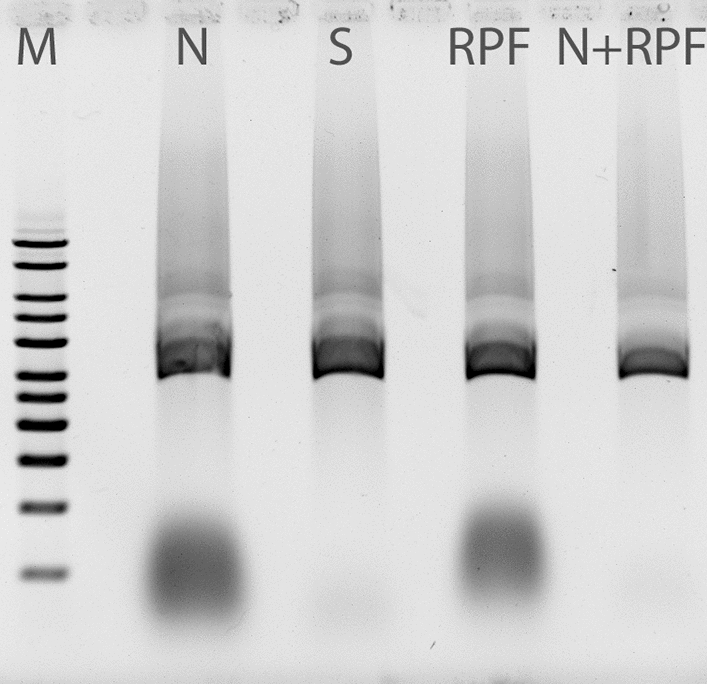
Agarose gel electrophoresis of RNA/DNA containing lysates for comparison of enzyme activities of bovine RNase A and RNase PF. N: lysis without addition of RNase, S: lysis with RNase A (Sigma-Aldrich) added to the resuspended cells. RPF: lysis with RNase PF added to the resuspended cells. N + RPF: cleared lysate from lysis N treated with RNase PF. M: DNA molecular size marker

The agarose gel electrophoresis in Fig. 6 shows that the lysis without addition of RNase results in a cleared lysate with a distinct RNA fluorescence. The addition of a commercially available bovine RNase A (Sigma-Aldrich) to the resuspended cells markedly removes the RNA as seen in the cleared lysate whereas in the lane for RNase PF (RPF) the RNA is unaffected. Since the same amount of enzyme activity was added to both the samples, this pointed to the possibility that the alkaline lysis conditions were denaturing the enzyme RNase PF far more than RNase A. This hypothesis is further strengthened by the fact that the RNA in the cleared lysate from the control lysis, could be efficiently removed by RNase PF. This meant that it was necessary to adjust the conventional protocol in such a way that RNase PF was added after the generation of cleared lysate instead of adding at the stage of cell resuspension. These initial application results also showed that the expressed RNase was free from DNase activity.

### RNase PF in plasmid production using anion-exchange chromatography

**Fig. 7:**
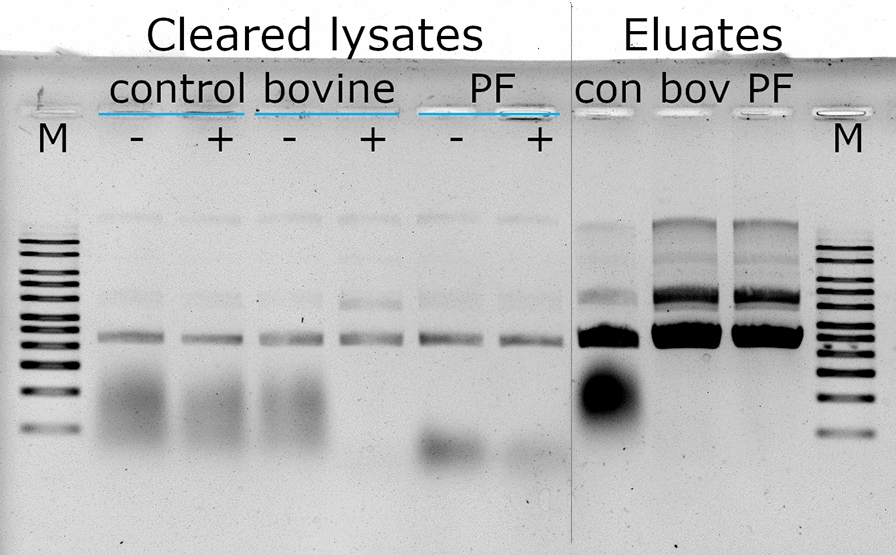
Application of RNase PF in a plasmid purification process. Cleared alkaline lysates without or with added RNase (bovine RNase A or RNase PF) were compared for removal of the RNA cloud. The lysates were then purified by a proprietary DNA purification chromatography step. The plasmid eluates from the three cases were compared on the basis of residual RNA. – and + refer to before and after treatment with RNase and incubation at 37 °C for 15 min. For control, no RNase was added

In the first trial, the effective biomass lysed was 13 g. The cleared lysates are shown in the left part of Fig. 7. In the lanes of the negative control, the cloud of RNA in the lower molecular weight region can be identified. Upon addition of bovine RNase A and the incubation of the sample for 15 min at 37 °C, a complete degradation of the RNA can be seen. This effect was somewhat less pronounced in the case of RNase PF, nevertheless the RNA content reduced to such an extent that subsequent purification by anion exchange chromatography was possible. From the chromatography, the eluted fractions loaded on the gel (eluates) showed that the control was heavily contaminated with RNA which bound to the chromatography medium and eluted along with the plasmid. In the case of bovine RNase A as well as RNase PF the elution fractions gave pure plasmid DNA.

**Fig. 8:**
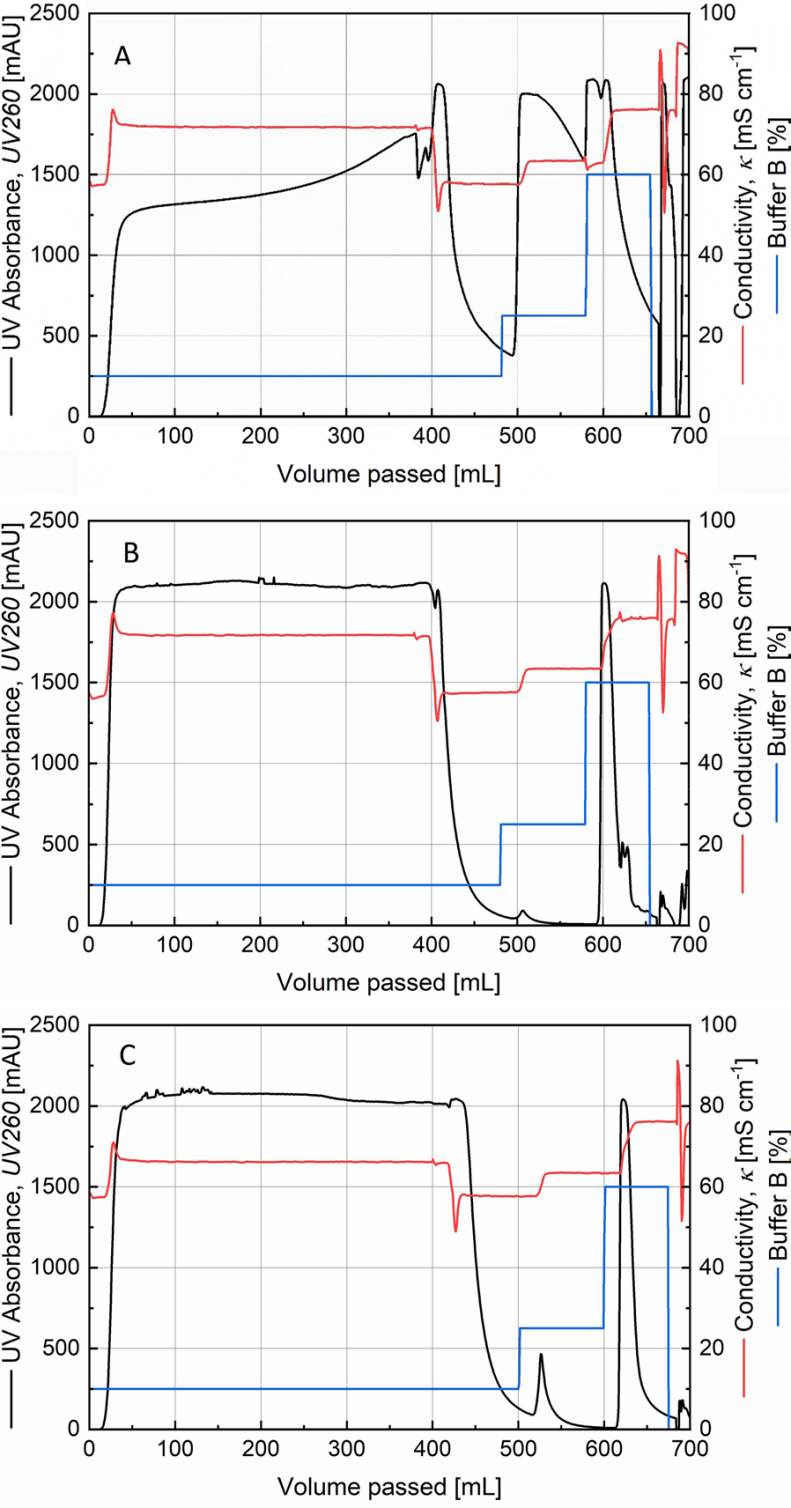
Chromatograms from a plasmid purification process without addition of RNases (**A**), addition of bovine RNase (**B**) or addition of RNase PF (**C**) to the cleared alkaline lysate feed. The eluates at 60 % elution buffer in the case of B and C are sharp peaks corresponding to pure plasmids as seen in Fig. 7

The chromatograms from the DNA purification in each case enable a very good understanding of the effects of RNA contamination (Fig. 8 A–C). In the control with no added enzyme, the RNA competes very effectively for binding sites causing a steady increase in UV260 absorption in the flowthrough and eventual breakthrough. As a direct consequence, there is a very prominent release of bound molecules during the wash with 25 % buffer B. The elution peaks with 60 % buffer B are irregular and point to a mixture of different molecules eluting together. This is confirmed in the agarose gel image in Fig. 7. In the case of bovine RNase A and RNase PF, the flowthrough curves are stable, the wash peaks are relatively small and the elution peak sharp, pointing to a pure single type of molecule being eluted out which is again confirmed in the agarose gel image to be the plasmid DNA.

**Fig. 9:**
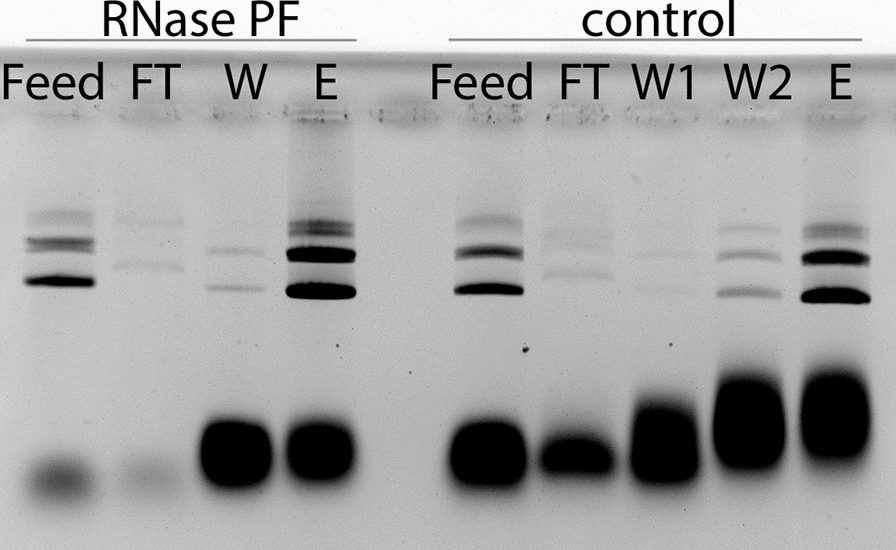
Agarose gel analysis of different fractions from a plasmid purification process by anion exchange chromatography after lysis of 50 g biomass each with addition of RNase PF to the cleared alkaline lysate or without (control). FT: Flowthrough, W: Wash, E: Elution

Upon scaling up to a lysis of 50 g biomass, it was found that the current level of volumetric activity in the RNase PF enzyme preparation was insufficient. An agarose gel analysis showed that RNA degradation took place but not to completion (Feed in Fig. 9). Subsequent anion exchange chromatography of the cleared lysates showed what this would mean for the plasmid downstream purification procedure (Fig. 9). In the case of RNase PF, the feed into the Äkta was clearly low in RNA content but nevertheless the residual RNA bound to the column. This is evident in the wash fraction and finally in the eluted product where the plasmid is contaminated with the residual RNA. In the case of control without enzyme addition, the feed and the flowthrough have larger amounts of RNA, the wash was collected in two fractions both heavily contaminated with RNA and the final product had more RNA than in the case of RNase PF.

To summarize, the degradation was not sufficient to result in a clean chromatography product and this can be traced back to the low volumetric activity of the enzyme preparation which sets the limit for the current capability for application.

## Discussion

RNA is a major competitor during the chromatographic purification of plasmid DNA. Strategies to reduce RNA content of cleared lysate feed improve the success of the chromatography step by allowing higher binding of the target molecule [12]. The experiments described in the current work are an attempt to develop a recombinant RNase that would allow an efficient purification of plasmid DNA while avoiding the quality concerns of using bovine RNase A.

Recombinant expression of the *B. amyloliquefaciens* RNase in high yields in *E. coli* has been known to be very challenging. As long as barnase is expressed in a native and active form, all previous studies have reported the necessity of co-expressing the intracellular inhibitor protein barstar. A strategy for periplasmic targeting using the PhoA signal sequence is presented in the current work. This should act as a safety measure against toxicity, whereas any intracellular barnase that has not been transported should be neutralized by the inhibitor. Previously, Voss et al. [12], cloned for periplasmic expression but the PelB leader sequence was found to be unsuitable for this case and therefore cytoplasmic fraction was focused upon in their work.

Low-level expression of BRP causes a semi-selective increase in outer membrane permeability which results in the release of periplasmic proteins into the extracellular medium [16, 17]. The *kil* gene of pColE1 coding for BRP was co-expressed under the control of a weak growth-phase-dependent promoter of the *fic* gene. This would allow regulation of strength, time and automatic BRP activation for extracellular expression while avoiding quasi-lysis and lethality. The advantage with extracellular expression is not only the prevention of accumulation of toxic proteins in the cell but also an improvement over periplasmic / cytoplasmic expression in that there are no extra lysis steps involved and specific target protein concentration is higher. On the other side, extracellular expression dilutes the enzyme and the challenge would be to handle large volumes of fluid during downstream processing.

One method of improving the efficiency of the RNA degradation during plasmid purification even with a limited amount of recombinant RNase is to vary the time of resuspension of cells in a Tris-EDTA buffer prior to alkaline lysis. We have observed that *E. coli* native enzymes can be active in a way that has not been extensively characterized. In other experiments, longer resuspension times led to reduced RNA levels while also generally increasing different forms of plasmid DNA instead of the desired ccc form (data not shown). Continuously decreasing fluorescence of RNA bands with respect to resuspension time can be shown in an agarose gel, which could be better studied in order to set up a combination strategy with added recombinant RNase. The effect of endogenous RNase activities at the stage of cleared lysate (through enzymes that survived the alkaline lysis process) was harnessed to increase the resulting plasmid purity [18]. However, a collateral damage to the plasmid DNA was also clearly observed and therefore this strategy would have to be optimized to ensure reproducibility. It must be noted that long incubation times are not compatible with an efficient process for large scale plasmid production. Conversion of ccc form into the oc form would lead to general loss of useful plasmid DNA.

Barnase has a pI of 9.2 [19] and cation exchange chromatography with a buffer pH of 5 was decided to be used as the primary method of purification for RNase PF. This also has the advantage that any contaminating DNA molecules could be separated away. For a cation exchanger, binding of the target enzyme onto the SP Sepharose Fast Flow column was not possible at a conductivity of 18 mS cm^− 1^. With the current nutrient medium, the cell-free supernatant from a fermentation broth was also found to have a conductivity of 12 mS cm^− 1^. In our experiments, the conductivity had to be reduced down to 4 mS cm^− 1^ for a successful binding to the column.

Ammonium sulphate precipitation as the immediate concentration step offered the possibility to use resuspended salt-rich proteins directly into the chromatography and thus develop HIC as a capture step. If the process is to be scaled up then ammonium sulphate would not be an ideal choice to use in large quantities since it is harmful to the environment. Since the ammonium sulphate step did not result in a significant reduction of volume and since it was not possible to bypass the diafiltration steps when going for cation exchange chromatography, ammonium sulphate precipitation was abandoned and the process was developed to take the final batch supernatant directly to tangential flow filtration. To ease the diafiltration step, salt-tolerant cation exchange chromatography media could be employed which would allow binding of the target protein at a relatively higher conductivity.

To avoid DNA contamination during the initial stages of the purification, the culture supernatant could be first acidified. Acidification of culture broth prior to cell separation was described by [8]. In periplasmic expression this is also expected to release periplasmic proteins [20].

From the SDS-PAGE image of Fig. 5, it is seen that there are still other host cell proteins present in the chromatography eluate. Using centrifugal filters with a cutoff of for example 30 kDa, it should be possible to remove most of the higher molecular weight proteins. Furthermore, the purified RNase is to be applied to cleared lysate from an alkaline lysis for the plasmid preparation. Although this cleared lysate is reduced in its protein content, it still contains proteins which also originated from *E. coli*. The chromatographic steps for plasmid purification have been shown to be very effective in drastically reducing the host cell protein content in the plasmid preparation.

### A note on enzyme activity

A maximum of 4 KU mL^− 1^ could be measured in the enzyme preparations in this study. Cell-free supernatant from 10 L fermentation broth resulted in about 210 KU. Literature reports based on a different promoter for expression, a different subcellular location of the product and therefore a different purification strategy give a value of 510 KU that could be achieved [12].

There is a difficulty in comparing literature reports because another method of enzyme activity measurement based on increase of absorbance at 260 nm is also used. A method of comparison for practical purposes would be to describe the enzyme activity in terms of the amount of bacterial biomass that could be processed with it for plasmid production—normally, bovine RNase is added to resuspended cells in excess for overdigestion and to get through the lysis step.

When calculating the amount of enzyme to add to cleared lysate, the level of host cell RNA is highly variable in each cleared lysate which leads to non-reproducible conditions. The use of native enzyme activity by elongated resuspension time is not exactly controllable. Fluorescence based measurement of RNA levels in cleared lysates are not reliable and therefore a better method of RNA quantification is based on peak area in a High Pressure Liquid Chromatography. Following this method, Kunitz units of enzyme activity that are needed to degrade a definite amount of RNA by following peak reduction would be needed. This would help in standardizing processes and in the judicious use of recombinant enzymes.

## Conclusions

RNase PF was expressed recombinantly in *E. coli* and purified from the culture supernatant. Its applicability for plasmid DNA purification was shown whereby it was necessary to adjust the procedure to allow the addition of the recombinant enzyme at the stage of cleared alkaline lysate. Summarizing all our results, there was a clear necessity to increase the specific productivity of the enzyme if this process was to be meaningfully scaled up. To this end, we recently engineered the expression plasmid towards the T7 promoter system. After experiencing difficulties with the corresponding expression strain due to extreme leaky promoter activity, an optimal expression strain was found which allowed controlled RNase PF expression. To continue this work, it needs to be assessed if the T7-based expression can improve the specific enzyme productivity to such a level that would result in an increase in volumetric activity of the enzyme preparations by at least a hundred-fold. The aim would then be to meaningfully aid in the lysis of 50 g of bacterial biomass for plasmid preparation as described above.

## Methods

### Host Strain and plasmid

*E. coli* DH5α was used throughout the study for cloning the recombinant barnase gene as well as testing its expression.

### Construction of plasmid PF1680

Firstly, PF1650 containing a fragment with a promoterless barnase gene and a complete barstar expression unit was synthesized (Eurofins GmbH). To clone a promoter upstream of the barnase gene, a 1.3 kb fragment from plasmid pP11 (PlasmidFactory GmbH & Co. KG) containing the P_BAD_ promoter and the *araC* gene was isolated by restriction with EcoRI, HindIII and NotI. PF1650 was restricted with EcoRI and HindIII and the resulting 4468 bp fragment was isolated and dephosphorylated with FastAP. The two fragments were ligated at the EcoRI and HindIII ends. The resulting plasmid was termed PF1680 (Additional file 1).

### Shake-flask cultivation

Experiments were carried out in 250 mL working volume in 1 L shake flasks with baffles in a complex medium. The inoculum was 1 mL of a glycerol stock of a verified clone. Ampicillin was added to a final concentration of 100 µg mL^-1^ to select for plasmid-containing cells. The cultivation was tested at temperatures of 30 and 37 °C. Shaking was carried out at 120 rpm and 2 cm deflection in horizontal orbital shakers.

The cultures were induced with L-arabinose (final concentration 10 g L^-1^) at the end of exponential growth phase.

### Fermentation

Batch fermentation was performed in a fermenter (Bioengineering) with a working volume of 10 L. The fermenter was inoculated from a shake-flask grown preculture at a 1 % v/v basis. Ampicillin (100 µg mL^-1^) was used only in the preculture. An aeration rate of 1 vvm, initial impeller speed of 200 rpm, pH of 7.0 and temperature of 37 °C were the conditions to be controlled. Dissolved oxygen concentration was cascaded to impeller speed to be maintained at a minimum of 60 % saturation. At 6 h of fermentation time, the culture was induced with L-arabinose at a final concentration of 10 g L^-1^. A scale-up fermentation in 20 L volume was performed in a Biostat CPlus fermenter (Sartorius) with similar operating conditions.

### Downstream processing

Cells in the culture broth were separated in a 1–16 K centrifuge (Sigma) for 10 min at 8000 ×*g*. Initially, ammonium sulphate at up to 60 % saturation was used to precipitate the proteins in the final supernatant from the batch cultivation after harvest. Overnight storage at 4 °C resulted in settled proteins. These proteins were centrifuged at 8000 ×*g* for 10 min at 4 °C and resuspended in 10 mM sodium acetate buffer (pH 5.1). Conductivity was measured using a WTW Tetracon 325 probe (WTW GmbH). For desalting through classical dialysis, a ZelluTrans/Roth® cellulose membrane with MWCO of 3.5 kDa (Carl Roth) was used. For 100 mL of sample, 19 cm of tubing was used and dialysed against 4.5 L of distilled water by slow overnight stirring. Desalting experiments were also carried out by ultrafiltration in a tangential flow filtration process for volumes upto 500 mL with the Pellicon® XL Cassette containing a 50 cm^2^ Biomax® Membrane (Merck Millipore) or the Vivaflow 200 with a 200 cm^2^ polyethersulfone membrane (Sartorius) both with a MWCO of 5 kDa. For a scaled-up process, the protein solution was pre-filtered through a 5 μm mdi ClariCapPP polypropylene capsule filter (Advanced Microdevices). A Quattroflow pump system for TFF was used in combination with an Omega Centramate membrane (OS005T12, PALL) that had a MWCO of 5 kDa and a surface area of 0.1 m^2^.

### Cation exchange chromatography

The retentate from the ultrafiltration was purified using the SP Sepharose Fast Flow (GE Healthcare Life Sciences) resin in a Äkta purifier chromatography system. Equilibration buffer (Buffer A or A1) was 0.1 M or 0.01 M sodium acetate/ acetic acid buffer (pH 5.0). Elution buffer (Buffer B) contained 1 M NaCl, 100 mM Tris and 10 mM EDTA (pH 7). The enzyme was eluted using a salt gradient of 0 to 100 % Buffer B over 1 h or through step-wise increases to 25 %, 50 and 100 % Buffer B. Cleaning-in-Place was done using 0.5 M NaOH through the A2 inlet of pump A.

### Scale up of SP Sepharose FF

In order to process large volumes at a relatively higher flow rate, scaling up of the chromatography column was implemented as shown in Table 3. The main guiding conditions are that column diameter is to be increased to accommodate more material, volumetric flow rate is to be increased so as to maintain the same linear velocity through the column. For ease of operation, the flow rate with the XK26 column was rounded up to 10 mL min^− 1^.

**Table 3:** Scale up of SP Sepharose FastFlow column

Parameter	Small scale	Higher scale
Column hardware	Glass column 15 × 125, Kronlab	XK26, GE Healthcare
Radius	0.75 cm	1.3 cm
Bed volume	5 mL	20 mL
Bed height	2.9 cm	3.76 cm
Volumetric flow rate	3 mL min^− 1^	9.04 mL min^− 1^
Linear flow velocity	1.7 cm min^− 1^	1.7 cm min^− 1^

### Hydrophobic interaction chromatography

The resins Capto Phenyl High Sub (GE Healthcare) or Toyopearl 650 M-Butyl (Tosoh Bioscience) were packed into a XK26/20 column (GE Healthcare) to a bed volume of 25 mL and 23.7 mL respectively and operated with an Äkta purifier chromatography system for testing HIC as a secondary purification step. The column was equilibrated with a high salt buffer containing 2 M ammonium sulphate. The feed solution to the chromatography was mixed with 3 M ammonium sulphate (pH 5.3) to yield a final concentration of 2 M in the sample. The operational volumetric flow rates were 20 mL min^− 1^ for the Capto Phenyl column and 5 mL min^− 1^ for the Toyopearl column. The proteins were eluted against deionized water acting as ‘Buffer B’.

### DNA removal

The membrane adsorber Sartobind STIC PA (Sartorius) with a bed volume of 1 mL was used to remove contaminating DNA in purified and concentrated enzyme fractions from the previous chromatographic steps. For small volumes upto 20 mL a manual syringe was used to push the sample through the membrane adsorber. For larger volumes, the adsorber was connected to the Äkta purifier chromatography system and operated at 5 mL min^− 1^. Samples were taken for verification by qPCR using the kit FastStart Essential DNA Green Master (Roche). Primers were designed to amplify a 116 bp fragment in the beta lactamase gene in PF1680. The plasmid pUC21 containing an ampicillin resistance gene was used in known concentrations as a standard. Analysis was performed in a LightCycler 96 instrument (Roche).

### Assay to test for RNase activity

The substrate used for testing the enzyme activity was either a cleared lysate from plasmid isolation or yeast RNA from Roche (2.5 mg mL^-1^ in deionized water). Sodium phosphate buffers of concentration 100 mM and pH 6 or pH 7.5 were also suitable to dissolve the substrate.

To 15 µL of substrate, an equal volume of test sample or water as negative control was added in a 1.5 mL PP tube and incubated at 37 °C for 15 min. Following the reaction, the samples were mixed with Gel Loading Dye Purple 6X (New England Biolabs) and analyzed by agarose gel electrophoresis. Enzyme activities were visualized as disappearance of the fluorescence from the RNA bands. A 1 kb DNA molecular size marker from PlasmidFactory GmbH & Co. KG was run to check for release of plasmids.

### Enzyme activity quantification

RNase activity in purified samples was quantified based on the principle of Kunitz [21]. The assay was performed according to the protocol by Sigma-Aldrich [22] with only a change in the total reaction volume to 1.5 mL. The assay follows the degradation of yeast RNA by measuring the decrease in extinction at 300 nm.

### SDS PAGE

Samples were concentrated by centrifugal filters (UFC501008, Merck Millipore) with a molecular weight cut off of 10 kDa. Sample were then prepared by mixing with 4X loading buffer (containing 5 % (w/v) 2-mercaptoethanol and 2 % (w/v) SDS) and boiling for 5 min at 96 °C. Proteins were resolved in a 12 % polyacrylamide gel through electrophoresis in a Tris-glycine buffer. The molecular size marker used is PageRulerTM Prestained Protein Ladder (Thermo Fisher Scientific). The gels were washed for 15 min in deionized water and stained overnight with 0.02 % Coomassie Brilliant Blue G-250 (Bio-Rad) followed by destaining for 2 h in deionized water.

### Determination of total protein concentration

Total protein was estimated using a modified Bradford test kit (Carl Roth). The absorbances were measured at 595 nm and 450 nm and their ratio used to correlate with the protein concentration using bovine serum albumin as a standard. Measurements were carried out using Costar 3596 96-well cell culture cluster (Corning Incorporated) and a FilterMaxF5 Multimode Microplate Reader (Molecular Devices).

### Test of RNase activity in resuspended cells solution

A purified enzyme fraction of RNase PF containing 0.05 mg mL^− 1^ of protein was spectrophotometrically analysed and found to contain an activity of 2.6 Kunitz units mL^− 1^ (KU mL^− 1^). This is about 500 times lesser than a preparation made from commercially available bovine RNase A (Sigma-Aldrich). These two RNase preparations were compared in their effectivities to degrade RNA during alkaline lysis of bacteria. The enzyme preparations were added to the cells resuspended in resuspension buffer (25 mM Tris, 10 mM EDTA, 50 mM Glucose, pH 8.5).

The commercial RNase A contained 114 Kunitz units mg Protein^− 1^ which was only about twice the specific activity of the recombinant enzyme tested here. For the experiment, 250 mg biomass each were lysed either with 3.75 KU RNase A or 3.75 KU RNase PF. The cleared lysates were analysed by agarose gel electrophoresis. The cleared lysate from a biomass lysis without added RNase was taken as control. Furthermore, to 10 µL of this lysate, 10 µL of RNase PF was added and incubated at 37 °C for 15 min to test the activity of the enzyme at this stage.

### Application to DNA purification

Thawed biomass was lysed according to a protocol based on the original alkaline lysis method [23]. The cleared lysates were either treated with bovine RNase A or RNase PF and compared to a negative control without addition of RNases. Plasmid purifications were performed using a proprietary anion-exchange chromatography protocol using the Äkta purifier chromatography system. In the first experiment, 40 g of biomass was resuspended in 400 mL of 1X resuspension buffer. After proceeding with the lysis and neutralization buffers, the cleared lysate was produced by centrifugation at 9600 rpm for 7 min. The supernatant was filtered through a 0.2 μm mdi filter (Advanced Microdevices). This was split into three reaction tubes where the second and third tubes received bovine RNase A and RNase PF respectively. Therefore, effectively, 13.3 g of biomass was tested for each case. RNase activity assay was done as described above. In a subsequent scaled up experiment, 100 g of biomass was lysed and the resulting 3000 mL of cleared lysate was split into two reaction vessels. One served as control whereas the other received 100 mL RNase PF preparation with a volumetric activity of 1.5 KU mL^− 1^. Thus, the effective biomass amount tested here for application of RNase PF was 50 g.

## Supplementary Information

**Additional file 1.** Plasmid map for the RNase PF expression construct PF1680.

**Additional file 2.** Batch growth curve of DH5α-PF1680. Inlay: Assay for RNase activity in the extracellular fraction at corresponding time points (0–12 h) during the fermentation in 20 L scale. S: RNA, N: negative control. M: DNA Molecular size marker.

**Additional file 3.** Chromatogram from a Toyopearl 650 M Butyl hydrophobic interaction chromatography of a cell-free supernatant that was prepared for HIC by mixing with 3 M ammonium sulphate. Following the step-wise elution with deionized water in steps of 25%, 75% and 100% as Buffer B, 10% isopropanol (E8) was used for further elution. Inlay: RNase activity test of various fractions. S: RNA substrate, N: Negative control, F: cell-free supernatant from fermentation broth, F_d_: cell-free supernatant diluted, FT: Flowthrough fraction, E1–E8: Elution fractions. The high concentration of ammonium sulphate caused smearing and RNA band displacement in the elution fractions.

**Additional file 4.** RNase activity assay to test the suitability of Sartobind anion exchange membrane adsorber for the removal of residual plasmid DNA from purified enzyme fractions. S: RNA substrate, N: Negative control, En: purified enzyme fraction, FT: Flowthrough, W: Wash, E: Elution and C: CIP in Sartobind membrane adsorber. M: DNA molecular size marker.

## Data Availability

The data generated during this study represent proprietary information belonging to PlasmidFactory GmbH & Co. KG and are not publicly available. However, where it is possible, datasets can be made available by the corresponding author upon reasonable request.
