# Correction to: Recombinant expression of Barnase in *Escherichia coli* and its application in plasmid purifcation

**DOI:** 10.1186/s12934-021-01678-0

**Published:** 2021-09-27

**Authors:** Ram Shankar, Nina Schäfer, Marco Schmeer, Joe Max Risse, Karl Friehs, Martin Schleef

**Affiliations:** 1 PlasmidFactory GmbH & Co. KG, Meisenstrasse 96, 33607 Bielefeld, Germany; 2 Fermentation Engineering, Bielefeld University, Universitätsstrasse 25, 33615 Bielefeld, Germany

## Correction to: Microb Cell Fact (2021) 20:171 https://doi.org/10.1186/s12934-021-01642-y

Following publication of the original article [1], the authors would like to include the second corresponding author of the article. The second corresponding author is Martin Schleef and the affiliation of the author is given below,

Dr. Martin Schleef: PlasmidFactory GmbH & Co. KG and

Fermentation Engineering, Bielefeld University, Bielefeld, Germany. The original article has been revised.
