# Supplementary figures and images for: Recombinant expression of Barnase in Escherichia coli and its application in plasmid purification
Source: Microb Cell Fact. 2021 Aug 28;20:171. doi: 10.1186/s12934-021-01642-y (PMC8403359; doi:10.1186/s12934-021-01642-y)

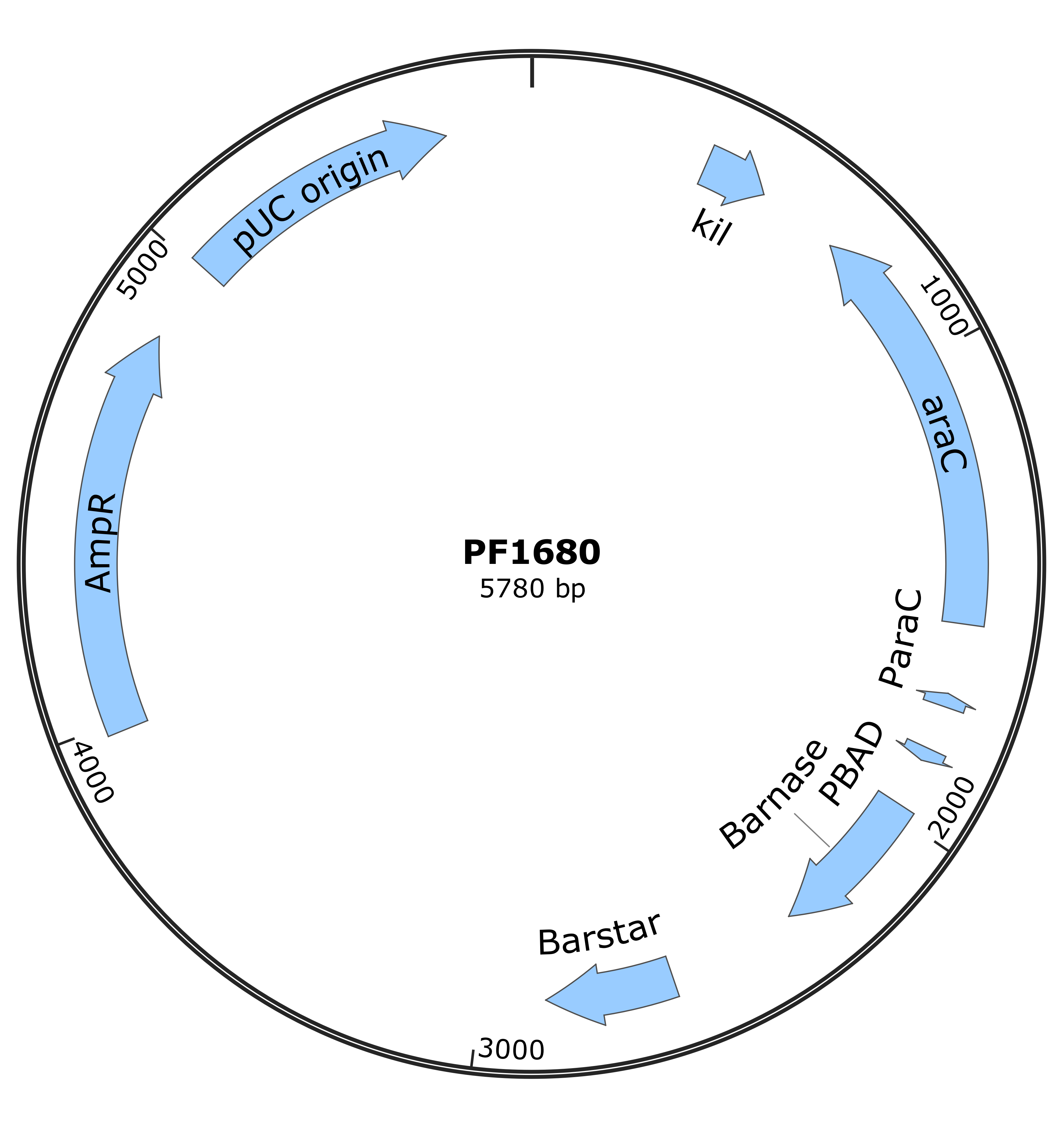

Supplement: Supplementary file 1 — Additional file 1. Plasmid map for the RNase PF expression construct PF1680. [file 12934_2021_1642_MOESM1_ESM.png]

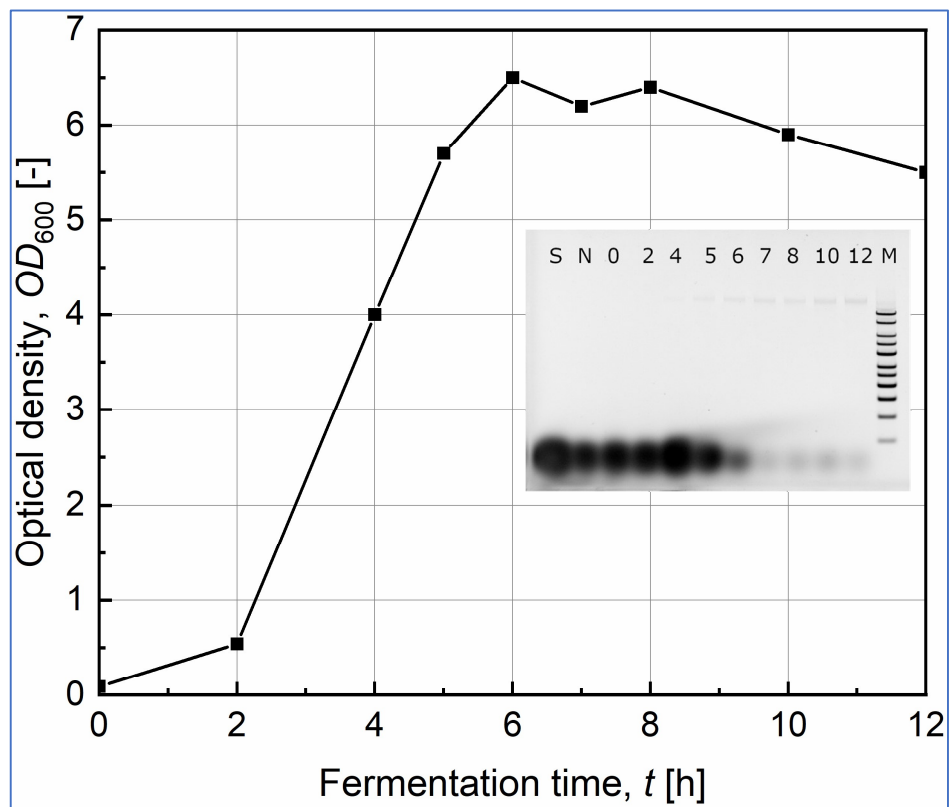

Supplement: Supplementary file 2 — Additional file 2. Batch growth curve of DH5α-PF1680. Inlay: Assay for RNase activity in the extracellular fraction at corresponding time points (0–12 h) during the fermentation in 20 L scale. S: RNA, N: negative control. M: DNA Molecular size marker. [file 12934_2021_1642_MOESM2_ESM.pdf]

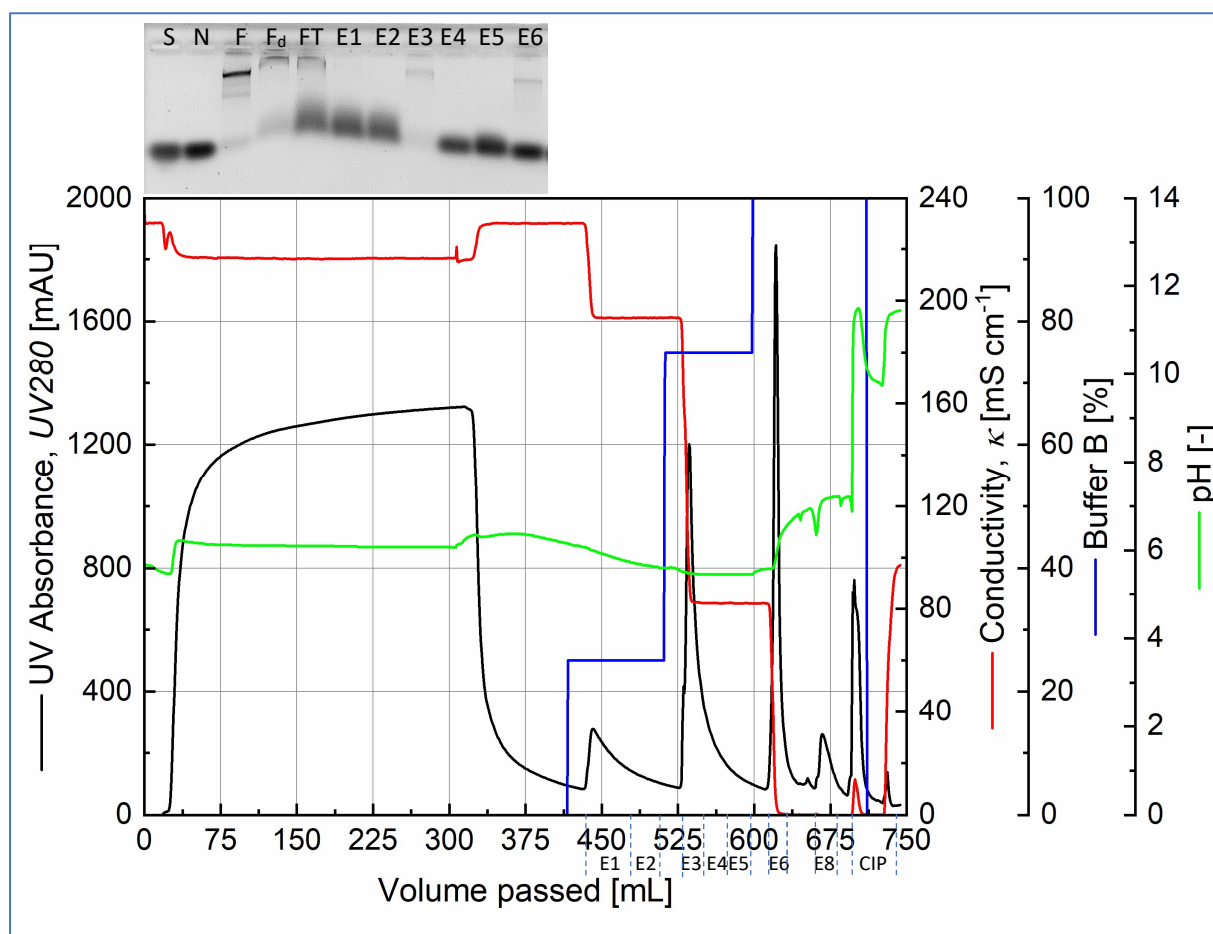

Supplement: Supplementary file 3 — Additional file 3. Chromatogram from a Toyopearl 650 M Butyl hydrophobic interaction chromatography of a cell-free supernatant that was prepared for HIC by mixing with 3 M ammonium sulphate. Following the step-wise elution with deionized water in steps of 25%, 75% and 100% as Buffer B, 10% isopropanol (E8) was used for further elution. Inlay: RNase activity test of various fractions. S: RNA substrate, N: Negative control, F: cell-free supernatant from fermentation broth, Fd: cell-free supernatant diluted, FT: Flowthrough fraction, E1–E8: Elution fractions. The high concentration of ammonium sulphate caused smearing and RNA band displacement in the elution fractions. [file 12934_2021_1642_MOESM3_ESM.pdf]

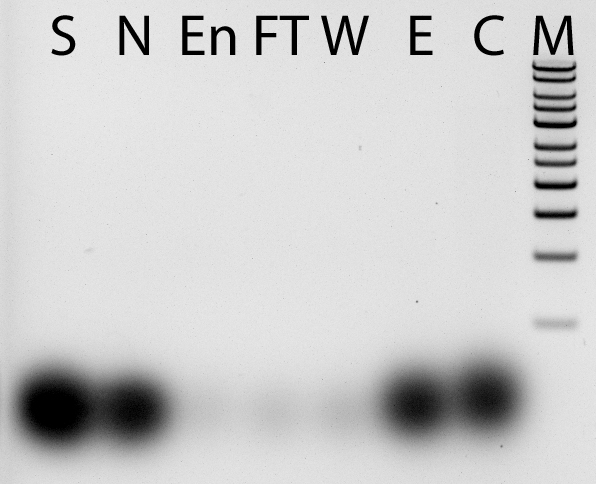

Supplement: Supplementary file 4 — Additional file 4. RNase activity assay to test the suitability of Sartobind anion exchange membrane adsorber for the removal of residual plasmid DNA from purified enzyme fractions. S: RNA substrate, N: Negative control, En: purified enzyme fraction, FT: Flowthrough, W: Wash, E: Elution and C: CIP in Sartobind membrane adsorber. M: DNA molecular size marker. [file 12934_2021_1642_MOESM4_ESM.jpg]
